# Higher Levels of Circulating Osteoprogenitor Cells Are Associated With Higher Bone Mineral Density and Lean Mass in Older Adults: A Cross‐Sectional Study

**DOI:** 10.1002/jbm4.10561

**Published:** 2021-10-17

**Authors:** Jack Feehan, Cassandra Smith, Nicholas Tripodi, Elizabeth Degabrielle, Ahmed Al Saedi, Sara Vogrin, Gustavo Duque, Itamar Levinger

**Affiliations:** ^1^ Department of Medicine – Western Health The University of Melbourne Melbourne VIC Australia; ^2^ Australian Institute for Musculoskeletal Science (AIMSS), Western Health The University of Melbourne and Victoria University Melbourne VIC Australia; ^3^ Institute for Health and Sport (IHES) Victoria University Melbourne VIC Australia

**Keywords:** STROMAL/STEM CELLS, CELLS OF BONE, OSTEOPOROSIS, DISEASES AND DISORDERS OF/RELATED TO BONE, AGING

## Abstract

Circulating osteo progenitor (COP) cells are a heterogeneous population of cells that circulate within the peripheral blood with characteristics of the bone marrow mesenchymal stem and progenitor pool. Little is known about the behavior of this cell population in humans. The aim of this study was to identify whether a relationship exists between COP cells (as a percentage of the peripheral blood monocytic cells) and musculoskeletal morphometry and to identify if COP have potential clinical utility as a biomarker for osteoporosis. We recruited 57 older adults (median age: 69 years; IQR: 65, 75 years) living independently in the community and performed cross‐sectional analysis to identify associations between the percentage of COP cells and body composition parameters, and through receiver operating characteristic analysis, we evaluated their ability to act as a biomarker of osteoporosis. COP cells were moderately associated with whole‐body bone mineral density (BMD) (*r* = 0.323, *p* = 0.014) and bone mineral content (BMC) (*r* = 0.387, *p* = 0.003), neck of femur BMD (*r* = 0.473, *p* < 0.001), and BMC (*r* = 0.461, *p* < 0.001) as well as appendicular lean mass (ALM) (*p* = 0.038) and male sex (*p* = 0.044) in univariable analysis. In multivariable analysis controlling for age, gender, height, and weight, COP cells remained strongly associated with neck of femur BMD (*p* = 0.001) and content (*p* = 0.003). COP cells were also a good predictor of osteoporosis (dual‐energy X‐ray absorptiometry [DXA] *T*‐score < −2.5) at the neck of femur (cutoff: 0.4%; sensitivity: 100%; specificity 79%) and total body (cutoff: 0.35%; sensitivity: 80%; specificity: 81%). This study shows strong relationships between bone parameters and COP cell number and male sex. They also have potential as a biomarker of osteoporosis, which may provide a new tool for advanced detection and screening in clinical settings. Future larger evaluation studies should verify the cutoffs for biomarker use, and further explore the relationship between COP cells and muscle. © 2021 The Authors. *JBMR Plus* published by Wiley Periodicals LLC on behalf of American Society for Bone and Mineral Research.

## Introduction

Ongoing maintenance of multiple body tissues requires recruitment, expansion, and proliferation of stem and progenitor cell populations, with the musculoskeletal system being no exception. Stem cell exhaustion and diminished regeneration are key pillars that underlie the modern concept of geroscience, which seeks to describe the biological mechanisms that drive age‐associated disease and loss of function.^(^ ^1^ ^)^ All stem cell and progenitor populations are thought to be vulnerable to these changes; however, it is perhaps most clearly characterized in the bone marrow mesenchymal progenitor cell (MPC) population.^(^ ^2^ ^)^ Age‐related deterioration of MPCs leads to reduced proliferation,^(^ ^3^ ^)^ diminished osteogenesis,^(^ ^4^ ^)^ and an increased tendency toward adipogenic differentiation,^(^ ^5^ ^)^ with detrimental effects on musculoskeletal health such as onset of frailty, osteoporosis, and sarcopenia.

More recently, a surrogate population of cells with similar characteristics to bone marrow MPCs have been identified in the circulation and consequently named circulating osteoprogenitor (COP) cells. COP cells are known to have the capacity for proliferation and mesodermal lineage differentiation,^(^ ^6^ ^)^ and high numbers are associated with some musculoskeletal disease states^(^ ^7^ ^)^ such as fracture,^(^ ^8^ ^)^ osteoporosis,^(^ ^9^ ^)^ and frailty.^(^ ^10^ ^)^ Although these cells may have the potential for clinical utilization, little is known about their relationship with bone formation or maintenance in vivo. Although these relationships with common musculoskeletal disease imply a connection with bone remodeling and maintenance, no direct evidence of this has been shown. These associations also raise the potential for COP cells to act as a biomarker for chronic musculoskeletal diseases; however, this is yet to be evaluated. Finally, there is also little evidence of how COP cells relate to body composition parameters other than bone, such as lean mass.

In this cross‐sectional study, we aimed to identify the relationship between COP cells and body composition in older adults living in the community. Additionally, we aimed to evaluate whether the number of COP cells in the circulation could act as a potential biomarker, discriminating those with low bone density from their healthy peers. We hypothesized that there would be associations between COP cells and bone parameters via bone densitometry, and that low levels would be predictive of osteoporosis in older adults.

## Subjects and Methods

### Study design and setting

This study is a cross‐sectional analysis, conducted at the Australian Institute of Musculoskeletal Sciences (AIMSS). This cross‐sectional analysis was performed using data from two ongoing interventional trials: the Wellderly project^(^ ^11^ ^)^ (Australia and New Zealand Clinical Trials Registry [ANZCTR] ref. 12618001756213), and Feehan et al, COP cells and Vitamin D supplementation^(^ ^12^ ^)^ studies (ANZCTR ref. 12619000685112). These studies had the same inclusion criteria and examined the percentage of COP cells via the same isolation protocol and means of assessment, making their baseline data compatible for pooled analysis. The participants were recruited from February 2018 to May 2020, with the two trials recruiting concurrently over that timeframe. Both studies were approved by the Melbourne Health human research ethics committee (Wellderly project reference: HREC/17/MH/335; COP‐VITD reference: HREC/45058/MH‐2018) and were conducted in accordance with the declaration of Helsinki. This study is reported in accordance with the Strengthening the Reporting of Observational Studies in Epidemiology (STROBE) statement guidelines.^(^ ^13^ ^)^

### Participants

Healthy community‐dwelling males and females aged ≥55 years were recruited for the study. Female volunteers were at least 12 months postmenopause (time since last menstrual period). Participants were excluded from the study if they had sustained a fracture or had begun osteoporotic medication in the preceding 3 months, had diabetes or were taking medications for glycemic control, had any hematological, myelodysplastic, or proliferative disorder, malignancy of bone, were on vitamin K or warfarin therapy, or who had a body mass index >40.0 kg/m^2^.

### Study assessments

#### Bone densitometry and body composition analysis

Dual‐energy X‐ray absorptiometry (DXA) was used to perform bone densitometry and body composition analysis. Imaging was performed with a Hologic Horizon DXA machine (Hologic Inc., Bedford, MA, USA) by an experienced radiographer. Total body bone mineral density (BMD), hip, lumbar spine, and wrist were assessed. In addition, fat and lean mass were assessed and appendicular lean mass by height squared (ALM/h^2^) was calculated automatically.

#### Peripheral blood mononuclear cell isolation

Blood samples were collected, in the morning following an overnight fast, into EDTA‐coated Vacutainers (BD Biosciences, Franklin Lakes, NJ, USA). Peripheral blood mononuclear cells (PBMCs) were immediately isolated, as described.^(^ ^12^, ^14^ ^)^ Briefly, peripheral blood samples were diluted 2:1 in sterile phosphate‐buffered saline (PBS) (Thermo Fisher Scientific, Waltham, MA, USA), and gently pipetted onto 10 mL of Ficoll‐Paque PLUS density gradient separation solution (Sigma Aldrich, St. Louis, MO, USA), in a 50‐mL conical tube, ensuring clear layer separation. This was centrifuged at 400*g* for 40 minutes with brakes off, with the resulting PBMC layer carefully aspirated, without collection of excess serum or Ficoll solution. The PBMCs were washed three times in PBS by centrifugation at 100*g* for 10 minutes to remove contaminant platelets, before being cryopreserved in 90% fetal bovine serum (FBS) (Sigma Aldrich, St. Louis, MO, USA), 10% dimethyl sulfoxide (DMSO) and stored at −80°C for batch analysis.

#### COP cell fluorescent labeling

When all samples were collected, they were prepared for batch analysis via immunofluorescent labeling and flow cytometry as described.^(^ ^15^ ^)^ The PBMCs were thawed and washed in fluorescence‐activated cell sorting (FACS) buffer (PBS with 5% FBS, 1mM EDTA) via centrifugation at 300*g* for 5 minutes. The PBMCs were resuspended in FACS buffer and incubated for 5 minutes with Fc receptor blocking reagent for 5 minutes at room temperature, before being incubated with an anti‐CD45‐fluorescein isothiocyanate (FITC) conjugated antibody (BD Biosciences, Franklin Lakes, NJ, USA) and a 780‐nm fluorescent fixable viability dye (BD Biosciences, Franklin Lakes, NJ, USA) for 30 minutes at 4°C in the dark. The PBMCs were then washed three times in FACS buffer, before undergoing fixation and permeabilization for staining of intracellular markers with the BD cytofix/cytoperm system as per manufacturer guidelines. Cells were incubated with 250 μL of paraformaldehyde‐based fixation/permeabilization buffer at 4°C in the dark for 20 minutes. They were then washed twice in a saponin‐based permeabilization/wash buffer before being resuspended in the same and incubated with an anti–osteocalcin‐phycoerythrin (OCN‐PE) conjugated antibody (BD Biosciences, Franklin Lakes, NJ, USA; 1:100 vol/vol) for 30 minutes at 4°C in the dark. Finally, cells are washed twice and resuspended in FACS buffer before proceeding immediately to flow cytometry. Fluorescence‐minus‐one (FMO) controls for each participant were prepared in tandem, through the same procedure minus the addition of the anti‐OCN‐PE dye. This ensured any changes in fluorescence due to the fixation and permeabilization were reflected in the controls.

#### Flow cytometric COP cell quantitation

COP cells were quantified as a percentage of the PBMCs (%COP) via multicolor flow cytometry. All samples were batch analyzed, to minimize the effect of intraday variability in the instrument. This analysis is reported according to the Minimum Information about a Flow Cytometry Experiment (MIFlowCyt) recommendations.^(^ ^16^ ^)^ All analysis was performed on a BD FACSCanto II flow cytometer, alongside BD FACSDiva software (version 8.0.1; BD Biosciences, Franklin Lakes, NJ, USA). Fluorescence optimization was performed with both single color and unstained compbeads (BD Biosciences, Franklin Lakes, NJ, USA), and PBMCs, to minimize light spillover. Photomultiplier tube (PMT) voltages were optimized with pilot samples, and once set, kept constant across samples. Unique FMO controls were used to set gating strategies for each participant, with region regions defined as expressing less than 0.01% of fluorescent events in the sample. A total of 30,000 events were analyzed for each sample, with the gating strategy described in Supplemental Fig. S1.

### Statistical analysis

Linear regression modeling was used to identify relationships between COP and each DXA variable of interest. COP data were log transformed prior to analysis, after which the fit was deemed adequate on inspection of residuals. Because of the logarithmic transformation, the results are expressed as exponentiated coefficients with 95% confidence intervals (CIs). Data was reported following a univariate analysis, between COP number and all other variables individually, as well as after multivariate analysis adjusted for age, sex, height, and weight. The alpha value was set as 0.05, with *p* values <0.05 being considered statistically significant. To evaluate the potential of COP cells for use as a biomarker, sensitivity and specificity were calculated for diagnosis of osteoporosis (*T*‐score ≤ −2.5) at the neck of femur, total body, and lumbar spine. Receiver operating characteristic (ROC) curves were plotted, and the area under the curve (AUC) was calculated to identify the discriminatory capability of COP cells in predicting osteoporosis. Finally, Youden's index was calculated to determine the optimal cutoffs to maximize sensitivity and specificity in each area.

## Results

### Population

A total of 57 individuals were included in this cross‐sectional study. Demographic characteristics and descriptive statistics of the cohort are shown in Table 1. The median age of the participants was 69 years (IQR 65, 75 years), with 70% (*n* = 40) of them being female. The median COP cell percentage was 0.56, within the range of previous studies.^(^ ^14^ ^)^

**Table 1:** Baseline Descriptive Statistics of the Cohort

Variable	Value
Subjects, *n*	57
Age (years), median (IQR)	69 (65, 75)
Sex, *n* (%)	
Female	40 (70%)
Male	17 (30%)
Morphological and body composition variables, median (IQR)	
Height (cm)	163.4 (158, 168.9)
Weight (kg)	75 (69, 85)
BMI (kg/m^2^)	28.3 (25.98, 31.19)
LS BMD (g/cm^2^)	1.001 (0.925, 1.0705) (*n* = 56)
FN BMD (g/cm^2^)	0.721 (0.665, 0.806) (*n* = 55)
Total BMD (g/cm^2^)	1.056 (0.985, 1.128)
% Body fat	38.4 (31.9, 45.4)
Lean/height (kg/m^2^)	16.8 (15.7, 18.3)
ALM (kg/m2)	6.77 (6.01, 7.54)
% COP	0.56 (0.36, 0.84)

ALM = appendicular lean mass; BMD = bone mineral density; BMI = body mass index; COP = circulating osteoprogenitor; IQR = interquartile range; NOF = neck of femur.

### Univariable analysis

**Fig 1:**
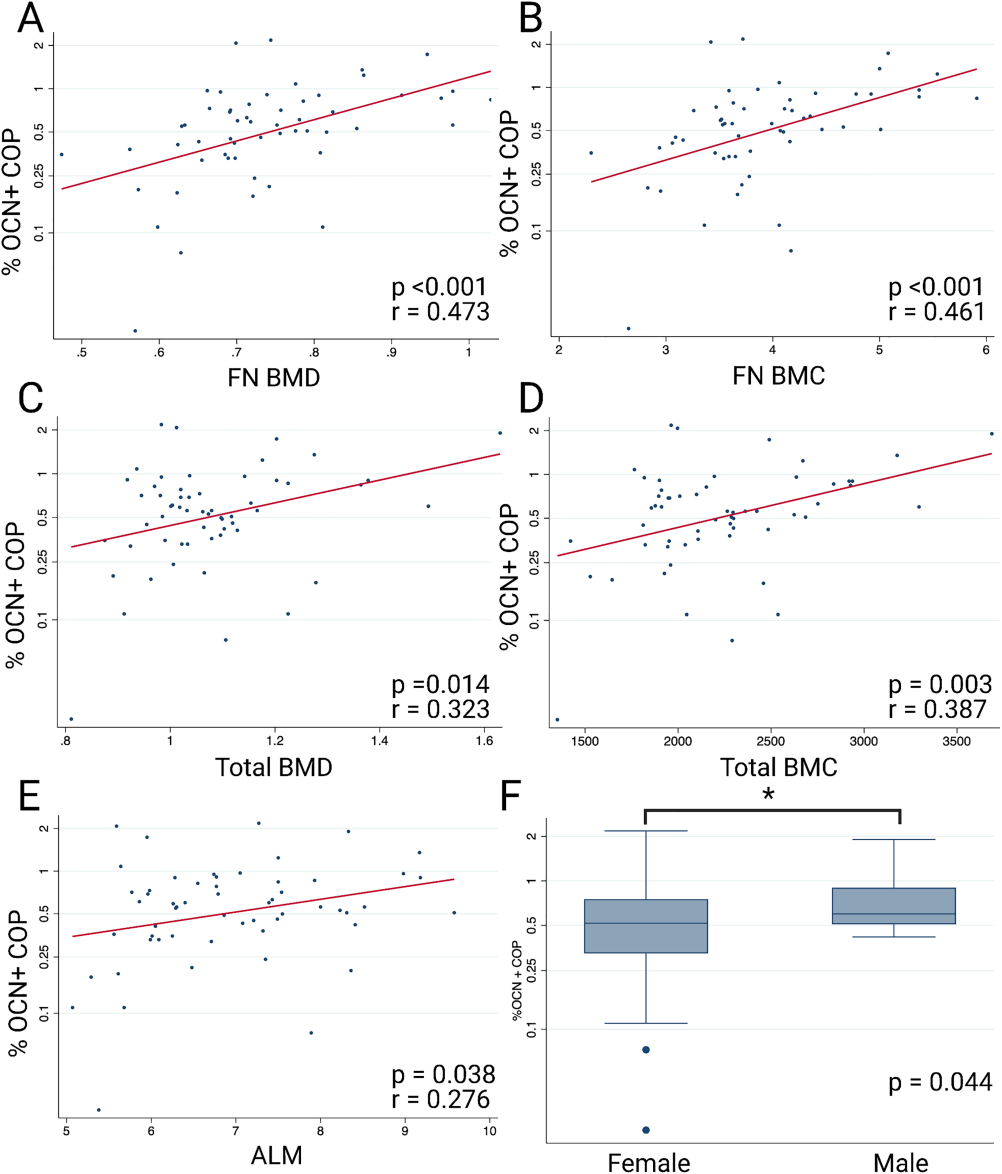
Univariable associations with COP cells. (*A*) COP versus femoral neck BMD, (B) COP versus femoral neck BMC, (*C*) COP versus total BMD, (D) COP versus total BMC, (*E*) COP versus ALM, (*F*) COP in males versus females.

A higher percentage of COP cells was moderately correlated with a higher total BMD (*r* = 0.323, *p* = <0.014) and bone mineral content (BMC) (*r* = 397, *p* = 0.003) (Table 2, Fig. 1C&D). An increase in total BMD of 0.1 kg/m^2^ was associated with a 20% increase in COP cells, and increasing total BMC by 10 g, correlated to a 1% increase in the percentage of COP cells. A higher percentage of COP cells was also moderately correlated with an increase in neck of femur (NOF) BMD (*r* = 0.473, *p* < 0.001) and BMC (*r* = 0.461, *p* < 0.001) (Table 2, Fig. 1A&B). For each increase of 0.1 kg/m^2^ in NOF BMD there was an associated increase in COP cell percentage of 40%, and for a 1‐g increase in BMC, there was a 65% increase in COP cells. COP cell percentage was also weakly correlated with a higher ALM (*r* = 0.276, *p* = 0.038, Fig. 1E), with a 1‐kg/m^2^ increase associated with a 23% increase in COP cells. Finally, males had a higher percentage of COP cells compared to women (*p* = 0.044, Fig.1F)), with male sex associated with a 61% increase. All other variables were not associated with the percentage of COP cells.

**Table 2:** Linear Regression Analyses

Variable	Univariable			Multivariable (adjusted for age, sex, height and weight)	
	Exponentiated coefficient (95% CI)	Correlation coefficient	*p*	Exponentiated coefficient (95% CI)	*p*
Age	1.00 (0.98–1.03)	0.048	0.726	N/a	
Height	1.02 (1.00–1.05)	0.236	0.077	1.01 (0.97–1.05)	0.752
Weight	1.02 (1.00–1.03)	0.237	0.076	1.01 (0.99–1.03)	0.353
BMI	1.03 (0.97–1.09)	0.123	0.364	N/a	
LS BMD (total) (increase of 0.1 kg/m^2^)	1.07 (0.95–1.22)	0.155	0.255	1.03 (0.90–1.18)	0.684
LS BMC	1.01 (1.00–1.02)	0.232	0.085	1.01 (0.99–1.02)	0.474
FN BMD (increase of 0.1 kg/m^2^)	1.40 (1.18–1.67)	0.473	<0.001*	1.41 (1.16–1.72)	0.001*
FN BMC	1.65 (1.26–2.15)	0.461	<0.001*	1.75 (1.22–2.51)	0.003*
Total BMD (increase of 0.1 kg/m^2^)	1.20 (1.04–1.38)	0.323	0.014*	1.16 (0.98–1.37)	0.089
Total BMC (increase of 10 g)	1.01 (1.00–1.01)	0.387	0.003*	1.01 (1.00–1.01)	0.06
Lean/Height (kg/m^2^)	1.09 (0.99–1.20)	0.232	0.083	1.00 (0.84–1.20)	0.98
ALM (kg/m^2^)	1.23 (1.01–1.49)	0.276	0.038*	1.10 (0.77–1.57)	0.585
Male versus female	1.61 (1.01–2.56)	N/a	0.044*	1.30 (0.66–2.55)	0.443

Results expressed as exponentiated coefficients which indicate the fold change in COP cells with each increase in unit of the comparison variable.

ALM = appendicular lean mass; BMD = bone mineral density; BMI = body mass index; CI = confidence interval; COP = circulating osteoprogenitor.

* Values are significant.

### Multivariable analysis

Once the linear regression model was adjusted for age, sex, height, and weight, the relationship of COP cells with BMD and BMC at the NOF remained significant (*p* = 0.001 and *p* = 0.003, respectively) (Table 2). When adjusted for covariables, an increase in BMD of 0.1 kg/m^2^ at the NOF was associated with a 41% increase in COP cells. Likewise, a 1‐g increase of BMC at the NOF was associated with a 75% increase in COP cells. All other variables were nonsignificant after adjustment for age, height, weight, and sex (Table 2).

### Preliminary assessment of COP cells as a potential biomarker for osteoporosis

**Fig 2:**
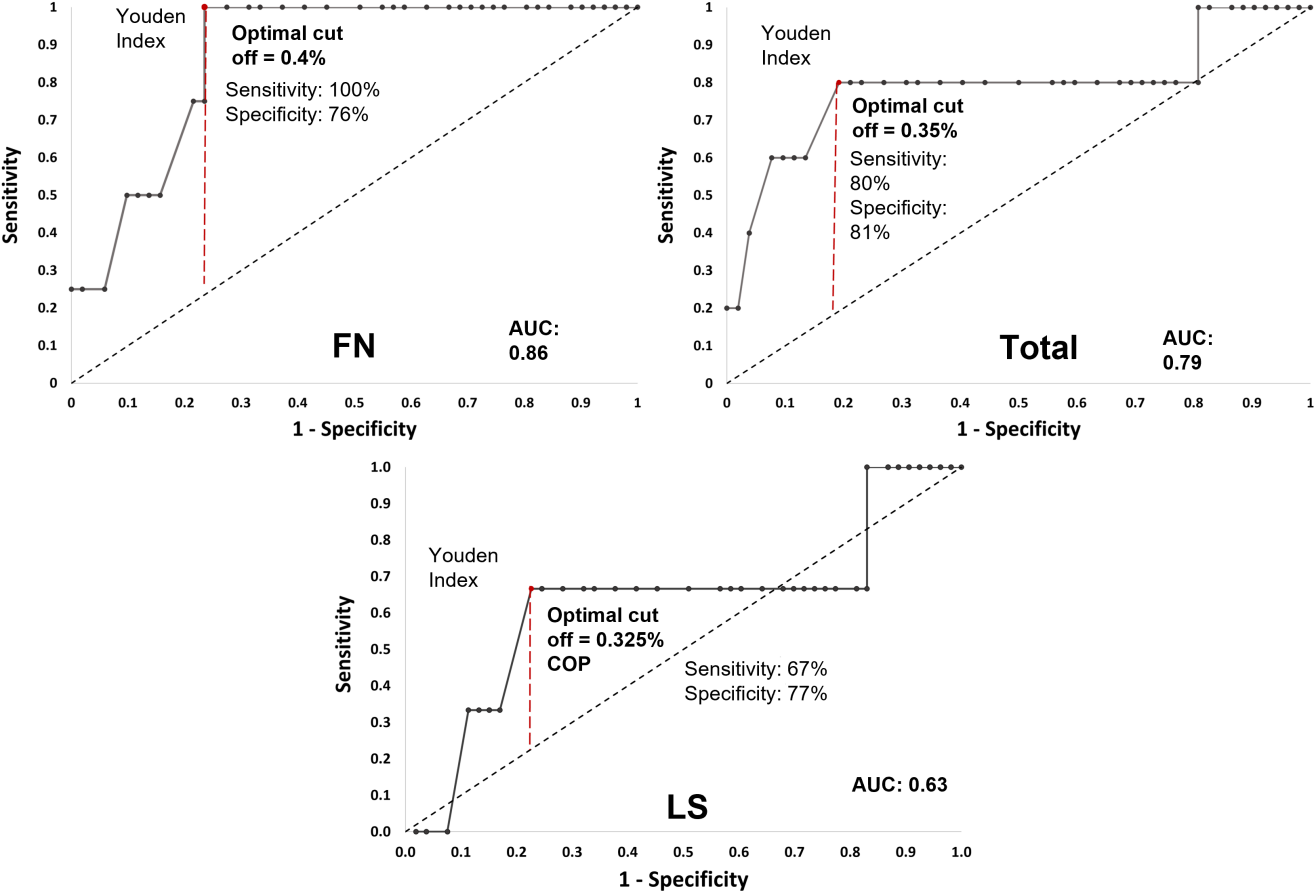
ROC curves for %COP as a biomarker of osteoporosis (*T*‐score ≤ −2.5). (Top left) Total body BMD. (Top right) Femoral neck BMD. (Bottom) Lumbar spine BMD. AUC = area under the ROC curve; ROC = receiver operating characteristic.

The sensitivity, specificity, and ROC curve analysis showed that COP cells had good diagnostic value in predicting total body and NOF osteoporosis (Fig. 2). Using the optimal cutoff of 0.35%, as identified by the maximum Youden J statistic, COP cells showed a sensitivity of 80% and a specificity of 81% in diagnosing total body osteoporosis, with an AUC of 0.79, indicating “good” diagnostic value. At the NOF, an optimal cutoff of 0.4% showed 100% sensitivity and 77% specificity, with an AUC of 0.86, again indicating a “good” diagnostic value. COP cells were a “poor” diagnostic indicator of osteoporosis at the lumbar spine, with 67% sensitivity, 77% specificity, and an AUC of 0.63 at the optimal cutoff of 0.325%

## Discussion

We report that a higher percentage of COP cells is strongly associated with a higher total body BMD, BMC, and BMD at the NOF. They were also positively correlated with male sex and with ALM. As a proof of concept, we have shown that COP cells should be explored as a future biomarker to identify people at risk of whole‐body osteoporosis and NOF osteoporosis.

This study is the first to show that a higher percentage of COP cells is related to BMC and BMD in older individuals. Although the mechanisms underlying this relationship are still unclear, it has previously been reported that COP cells can migrate to sites of bone formation and deposit mineralized osteoid in, at least in animals.^(^ ^17^ ^)^ COP cells are increased in states of bone formation, such as fracture, heterotopic ossification, and pubertal bone growth,^(^ ^17^, ^18^ ^)^ suggesting they have a role in the formation or remodeling of bone. The fact that COP cells are consistently present in the circulation^(^ ^14^ ^)^ suggests that they have some role in the ongoing maintenance of bone, and perhaps also an unknown role outside the skeleton. However, whether this is through direct osteoblastogenesis and mineralization, or regulation of MPCs or osteoblasts requires further investigation. The lack of association with bone density in the lumbar spine raises additional questions regarding their physiology. It is unclear why there might be differences between their impact on the axial and appendicular skeletons; however, they may be due to mechanical loading patterns driving cell biology, anatomical, or vascular differences between the locations assessed by DXA, or simply an artifact of the small sample size of the present study.

In the current study, we have shown that COP cells are positively associated with lean mass. Although COP cells are traditionally associated with osteogenesis, this evidence of multilineage correlations is intriguing, and opens several avenues for further investigation. Others have shown that this population of COP cells can differentiate into muscle cells in vitro^(^ ^18^ ^)^; however, there is little additional evidence supporting a potential role in muscle physiology. One study has shown hematopoietic COP cells to increase in number after a 3‐month weight‐bearing and resistance training program in older osteoporotic females, which could be taken to imply a role in muscular regulation.^(^ ^19^ ^)^ However, given the well‐documented cross‐talk between bone and muscle, and the anabolic effects of resistance and weight‐bearing exercise on bone, it makes causative associations challenging to draw from this evidence alone.^(^ ^20^ ^)^ Further studies are required to explore the association between hematopoietic COP cells and muscle and identify other possible mechanistic evidence of a relationship external to their role in bone physiology.

The COP cell findings in this study are within the range of previous studies regarding their number, and in that there was no association with age.^(^ ^14^ ^)^ Interestingly, another study found no relationship between this population of COP cells and sex^(^ ^14^ ^)^; however, herein we report that male sex was associated with 44% more hematopoietic COP cells compared to females. This is potentially explained by the present study focusing only on an older age demographic, whereas the other previous study examined individuals of a larger age span. The association between the decreased percentage of COP cells and female sex in this study could also be reflective of the increased prevalence of low bone mass in this demographic, an effect potentially masked in the previous work.

Our study also showed preliminary evidence of a potential use of COP cells as a biomarker of osteoporosis. The good performance of COP cells by ROC assessment (sensitivity and specificity >75%, AUC >0.75)^(^ ^21^ ^)^ as a biomarker for osteoporosis of the total body and NOF support their potential future use in clinical settings to diagnose this condition and monitor treatment. Although larger, population‐scale studies are required to verify the cutoffs and associations, this preliminary evidence of their biomarker status shows them outperforming many other previously studied markers of bone remodeling, such as cross‐linked C‐telopeptide (CTX) and amino‐terminal propeptide of type 1 procollagen (P1NP).^(^ ^22^, ^23^ ^)^ Future larger, prospective, and cross‐sectional studies should incorporate analysis of COP cells, to verify and evaluate these findings.

Although the methodology and analysis of this study are robust, the sample size of this study is relatively small, which may limit the generalization of these study findings in other cohorts. Importantly, because the sex balance of this study was weighted toward females, the findings need to be verified in males to ensure appropriate generalization. Although the NOF associations with COP were very robust even after adjusting for age, sex, height, and weight, the other variables studies were influenced by these factors. Given the association with higher COP cells in males, this is likely primarily a factor of sex; however, the relatively low number of males in the cohort makes these relationships difficult to ascertain. In addition, a larger cohort and longitudinal studies are required to further confirm the potential for COP cells to be used as a clinical biomarker for osteoporosis.

## Conclusion

Novel approaches for the diagnosis and management of chronic musculoskeletal disease are vital in the face of an aging population and COP cells may be a promising candidate for future investigation in this field. Their associations with bone and lean mass and potential utility as a biomarker for osteoporosis provide new avenues for the development of diagnostic approaches to osteoporosis, and potentially other musculoskeletal diseases such as sarcopenia. Future larger studies should evaluate these relationships further, as well as exploring the behavior of COP cells in other disease states.

## AUTHOR CONTRIBUTIONS

**Jack Feehan:** conceptualization; formal analysis; investigation; data curation; project administration; visualization; writing ‐ original draft. **Cassandra Smith:** conceptualization; investigation; data curation; project administration; writing ‐ review and editing. **Nicholas Tripodi:** investigation; writing ‐ review and editing. **Elizabeth Degabrielle:** investigation. **Ahmed Al Saedi:** investigation. **Gustavo Duque:** conceptualization; supervision; funding acquisition; writing ‐ review and editing. **Itamar Levinger:** conceptualization; supervision; funding acquisition; writing ‐ review and editing.

## Disclosures

Jack Feehan, Cassandra Smith, Nicholas Tripodi, Elizabeth Degabriele, Ahmed Al Saedi, Sara Vogrin, Gustavo Duque, and Itamar Levinger have no conflict of interest to declare.

### PEER REVIEW

The peer review history for this article is available at https://publons.com/publon/10.1002/jbm4.10561.

## Supporting information


**Supplemental Fig. S1** Flow cytometry gating strategy used to identify COP cells.

## Data Availability

The data that support the findings of this study are available from the corresponding author upon reasonable request.
