# Supplementary figures and images for: Higher Levels of Circulating Osteoprogenitor Cells Are Associated With Higher Bone Mineral Density and Lean Mass in Older Adults: A Cross‐Sectional Study
Source: JBMR Plus. 2021 Oct 17;5(11):e10561. doi: 10.1002/jbm4.10561 (PMC8567483; doi:10.1002/jbm4.10561)

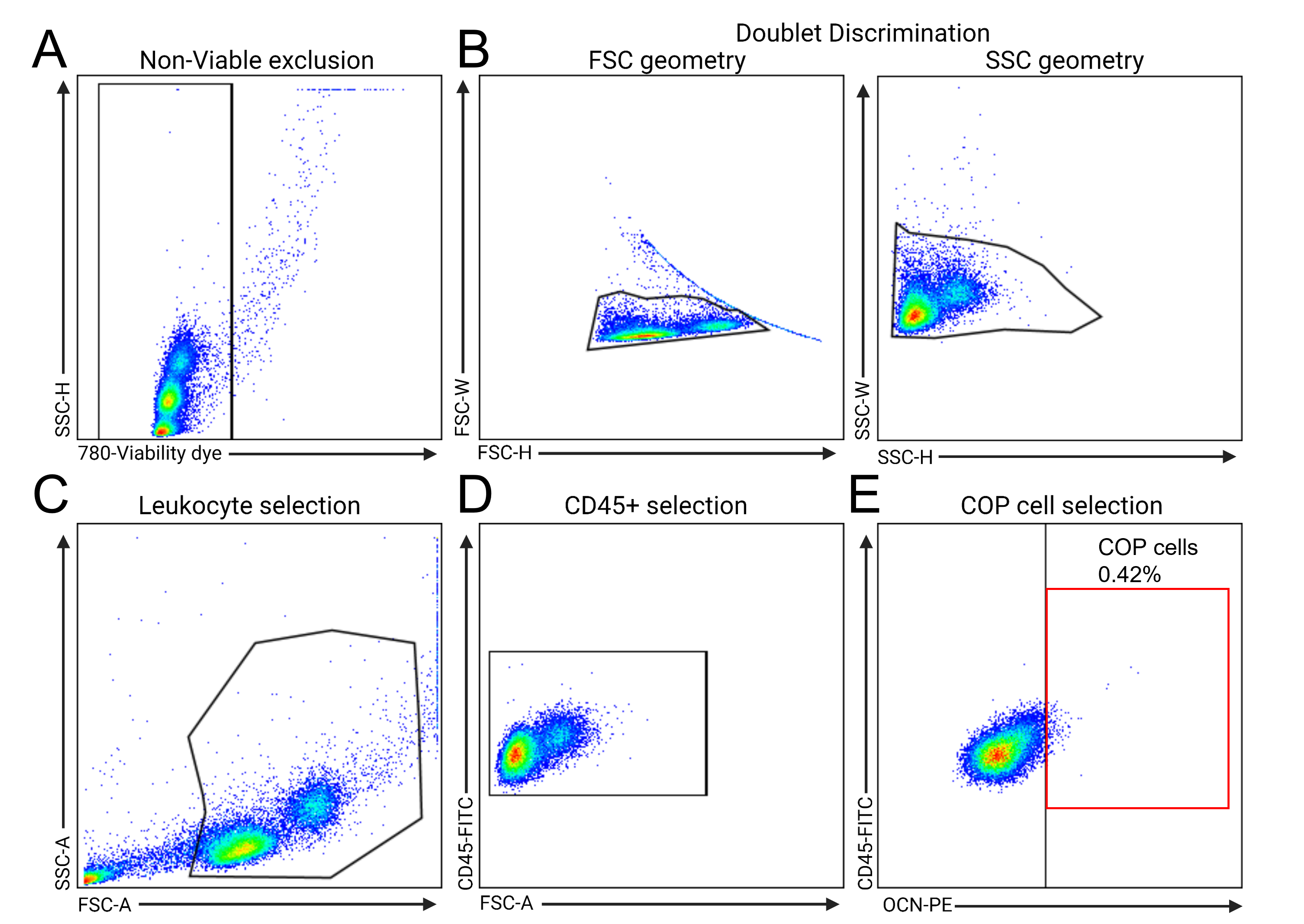

Supplement: Supplementary file 1 — Supplemental Fig. S1 Flow cytometry gating strategy used to identify COP cells. [file JBM4-5-e10561-s001.tif]
